# Metoprolol-induced Severe Liver Injury and Successful Management with Therapeutic Plasma Exchange

**DOI:** 10.7759/cureus.1209

**Published:** 2017-05-02

**Authors:** Cyriac Philips, Rajaguru Paramaguru, Pushpa Mahadevan, Jayasurya Ravindranath, Philip Augustine

**Affiliations:** 1 Hepatology and Liver Transplant Medicine, PVS Memorial Hospital; 2 Pathology, PVS Memorial Hospital; 3 Pathology, VPS Lakeshore Hospital, Kochi; 4 Nephrology, PVS Memorial Hospital; 5 Gastroenterology, PVS Institute of Digestive Diseases, PVS Memorial Hospital

**Keywords:** dili, idiosyncratic reaction, beta blockers, liver injury, hepatocellular injury, plasma exchange

## Abstract

Liver injury caused by metoprolol is very rare with current reports limited to an isolated elevation in transaminases. We report the first case of severe icteric liver injury leading to hepatic encephalopathy secondary to metoprolol use in a patient diagnosed with coronary heart disease. We also describe the histopathology of metoprolol-related liver injury, discuss mechanisms of injury with new insights on the immunological phenomenon, and shed light on the successful utility of early plasmapheresis as a salvage therapy in metoprolol-induced severe liver damage.

## Introduction

Metoprolol is a cardioselective beta blocker with potent activity against beta-1 adrenergic receptors with little activity against beta-2 adrenergic receptors. It is commonly approved for therapy in angina pectoris, coronary artery disease, acute myocardial infarction, and systemic hypertension. The usual dose in adult patients is 100 milligrams given in two to three divided doses. Common side effects include bradycardia, hypotension, fatigue, dizziness, insomnia, memory loss, depression, and impotence [1]. Metoprolol has been linked to only rare cases of drug-induced liver injury, with few reports of the clinically apparent acute liver injury. However, a severe icteric liver injury has not been reported in the literature [2]. We report the case of a middle-aged woman who was diagnosed with acute myocardial infarction and in whom eight weeks of metoprolol use led to severe acute liver injury, leading to jaundice and Grade 1 hepatic encephalopathy. Initial drug withdrawal resulted in mild improvement in hyperbilirubinemia. Persistence of encephalopathy warranted plasmapheresis sessions with substantial clinical improvement and biochemical normalization. 

## Case presentation

A 56-year-old woman, diagnosed with acute myocardial infarction on November 16, 2016, underwent angioplasty and stenting. She was started on an oral aspirin and clopidogrel combination to be taken daily once after lunch, atorvastatin - 20 mg once after dinner, ticagrelor - 90 mg two times after meals, and metoprolol - 25 mg in two divided doses, which were continued until December 7, 2016. After three weeks, due to myalgia, the atorvastatin was stopped while the other medications were continued. The dose of metoprolol was increased to 100 mg per day after that. On December 28, 2016, during the second follow-up, ticagrelor and clopidogrel were stopped, and metoprolol and aspirin were continued. On January 16, 2017, the patient complained of loss of appetite and well-being without associated fever, skin rashes, or joint pains. She started using an over-the-counter multivitamin syrup. However, on January 28, 2017, she noticed darkening of the urine. Yellowish discoloration of sclera developed three days later, for which she consulted a general physician who ordered liver function tests and blood investigations for acute viral hepatitis inclusive of A, E, and B. The latter were non-reactive, and following liver function tests showed progressive hyperbilirubinemia with elevated transaminases. She developed reversal of sleep pattern and drowsiness towards the later part of February 2017 and was subsequently referred to our center for further management on March 6, 2017. On admission, the patient was conscious and oriented to place and person but not to time. She was deeply icteric without asterixis, pallor, or pedal edema. Organomegaly was not appreciable, and free fluid was absent. Blood investigations for antinuclear antibody were weakly positive with the mitochondrial pattern at 1 in 40 titers. Antibodies to liver-kidney-microsomal type 1, liver soluble antigen, smooth muscle, Smith antigen, double-stranded deoxyribonucleic acid, Rho antigen, and La antigen were negative. Total serum immunoglobulin G levels were within normal range. Repeat investigations for viruses including hepatitis A and E, cytomegalovirus, Ebstein-Barr virus, parvovirus, and herpes simplex types 1 and 2 were negative, and viral hepatitis B and C including viral DNA and RNA, respectively, were also negative. Liver function tests, coagulation profile, and arterial ammonia values from baseline until the last follow-up are shown in Table 1.

**Table 1:** Blood Investigations Serial blood investigational parameters of the patient from baseline until follow-up. TB – total bilirubin (mg/dL); AST – aspartate transaminase (IU/L); ALT – alanine transaminase (IU/L); AP – alkaline phosphatase (IU/L); GGT – gamma-glutamyl transpeptidase (IU/L); INR – international normalized ratio; ammonia – arterial sample, in microgram/dL.

*Time Periods*	TB	AST	ALT	AP	GGT	INR	Ammonia
Baseline	1.2	42	31	98	78	0.8	
3 weeks	0.8	38	32	102	72	1.1	
6 - 7 weeks	1.1	40	41	106	85	0.9	
8 - 9 weeks	1.6	36	32	99	86	1	
10 – 12 weeks	5.4	206	542	203	108	1.33	
13 - 14 weeks	15.6	998	1,124	198	118	2.54	98
14 weeks	22.3	1,023	1,568	176	148	2.61	102
15 – 16 weeks	METOPROLOL STOPPED						
16 weeks	24.8	1,823	2,262	224	192	3.82	238
PLASMAPHERESIS	Session x 3 (daily)						
16 - 17 weeks	8.2	134	198	98	102	1.56	68
PLASMAPHERESIS	Session x 2 (alternate)						
17 - 18 weeks	3.9	72	56	88	98	1.18	52
Follow-up (10 days post-discharge)	2.2	66	72	94	104	1.13	

**Figure 1:**
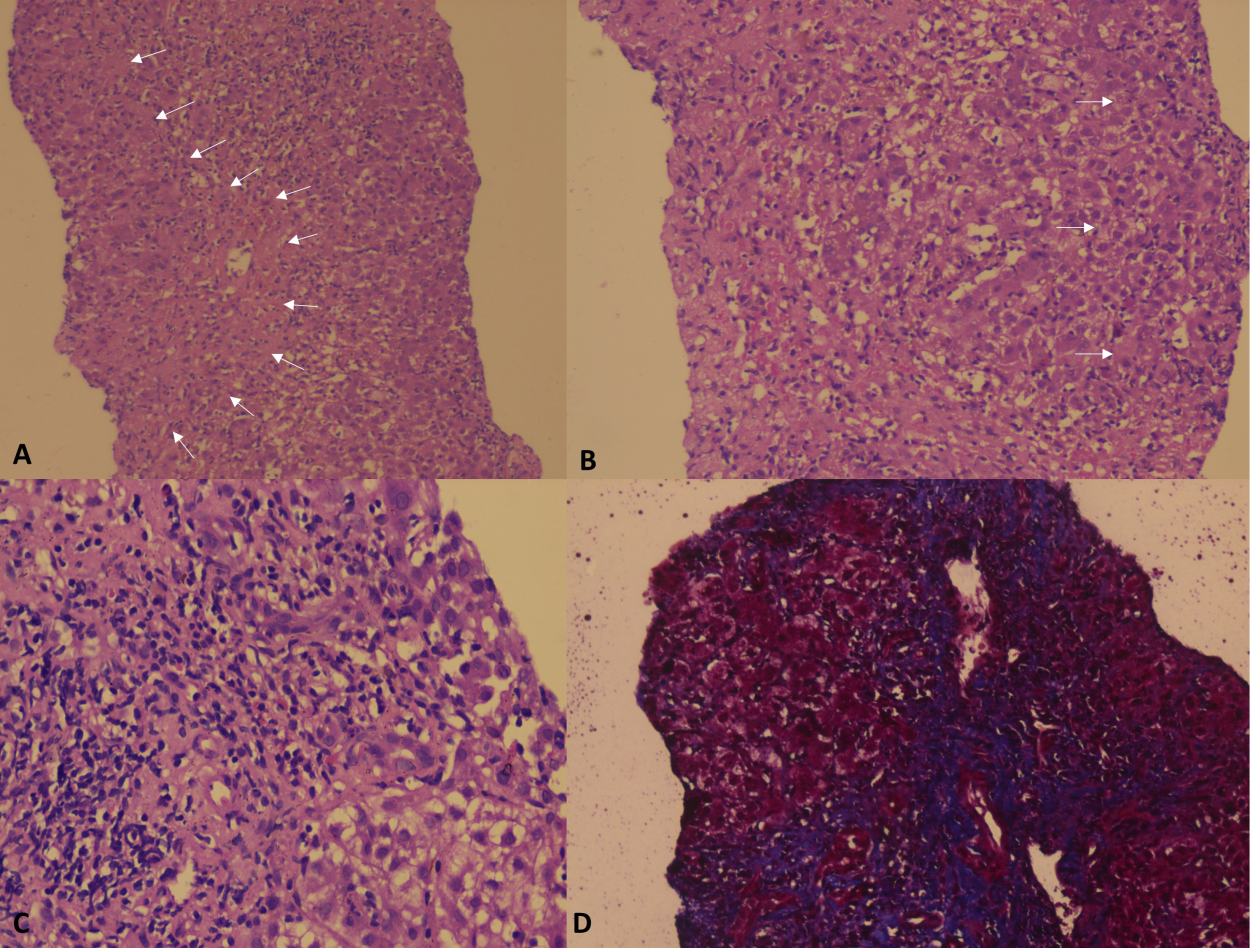
Liver histopathology Transjugular liver biopsy histopathology showing extensive bridging necrosis (A, arrows; 10X, Hematoxylin and Eosin stain) with surrounding normal hepatocyte islands (B, arrows; 20X, Hematoxylin and Eosin stain); mixed neutrophilic and lymphocytic inflammation (C; 40X, Hematoxylin and Eosin stain) and early fibrosis laying down at the site of necrosis (D, 40X, Masson's Trichrome stain).

In the presence of coagulation failure, a transjugular liver biopsy was performed. The liver histology showed the presence of extensive bridging necrosis with islands of normal surrounding hepatocytes and evidence of mixed inflammatory infiltrate predominantly composed of neutrophils and lymphocytes and occasional plasma cells with early fibrous laying down more within areas of necrosis (Figure 1).

**Figure 2:**
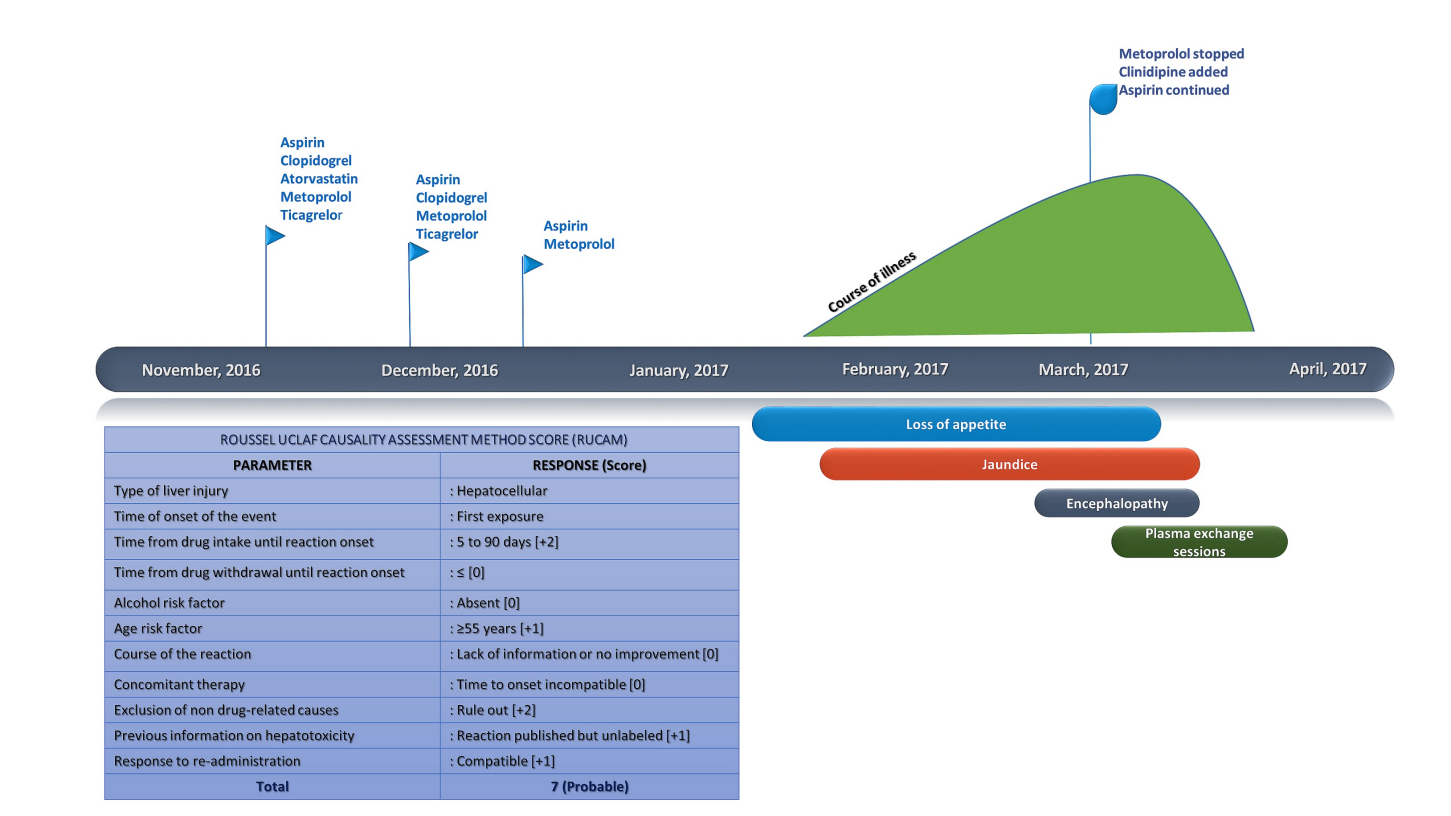
Clinical events and causality assessment Serial clinical events and Roussel Uclaf Causality Assessment Method score

Classical features of autoimmune hepatitis were not evident, and no inclusion bodies or granulomas in association with bile duct damage were noted; the findings were suggestive of acute hepatitis with bridging necrosis secondary to drug insult. A thorough review of drug history was undertaken, and based on the Roussel Uclaf Causality Assessment Method Score (RUCAM), "probable" drug-induced liver injury was attributed to the metoprolol [3]. The metoprolol was stopped immediately. Seventy-two hours after stopping metoprolol, the liver function only modestly worsened. However, hepatic encephalopathy increased in severity with excessive drowsiness and memory loss without asterixis. Given the severe drug-induced liver injury (DILI), therapeutic plasmapheresis through femoral line access using the membrane plasma separation technique was initiated. Single volume plasma exchange was done using standard hemodialysis equipment in high flux ultrafiltration mode without cytapheresis using the 4008S Classix and fx-Classix Filter (Fresenius Medical, Germany). One-third and two-thirds of plasma replacement were made with 5% human albumin and fresh frozen plasma, respectively. The volume of plasma to be removed was calculated using the formula, 0.65 × weight in kilogram × one minus hematocrit. Plasma exchange was done daily for the first three days with monitoring of hemogram, renal and liver biochemistries, serum electrolytes, arterial ammonia, and coagulation indices every 12 hours. Three sessions later, the patient showed improvement in hyperbilirubinemia, hyperammonemia, coagulopathy, and encephalopathy. Another two sessions were given on alternate days, totaling five in all. Following that, the liver function tests showed persistent improvement with progressive clinical well-being. As per our protocol for plasma exchange, we assess biochemical parameters after three initial sessions with the daily clinical assessment. Only in the event of worsening or new onset symptoms or signs during the course of the initial three sessions, do we perform interim biochemical assessments; hence, serial daily measurements were not made in our patient. The patient was on broad spectrum antibiotics with the addition of gram-positive coverage from the initiation of plasma exchange sessions, which is the standard operating protocol in our hospital. The patient did not develop infections in hospital, and baseline and protocol cultures were negative for bacterial or fungal growth. Serial lactate measurements were not performed in our patient as lactate monitoring during severe DILI is not a validated tool. In the presence of new onset sepsis, we perform lactate measurements as part of a blood gas analysis. Our patient did not develop fluid overload during plasma exchange sessions. We performed X-ray imaging of the chest only if there was definitive evidence of respiratory tract infection on daily clinical assessment or in the presence of new onset fever with chest symptoms, such as purulent sputum, tachypnea, or desaturation on room air. The patient was discharged two days later and on follow-up was found to have substantial improvement in clinical and investigational parameters with repeat antinuclear antibody testing showing negative results (Figure 2). 

## Discussion

Metoprolol is metabolized by three major oxidation pathways. Two of them, 0- dealkylation and alpha-hydroxylation, undergo genetically controlled polymorphisms - similar to debrisoquine, a derivative of guanidine, an antihypertensive drug similar to guanethidine, which is frequently used for phenotyping the CYP2D6 enzyme oxidation. Controlled studies have shown that the pharmacokinetic and pharmacodynamics of metoprolol are mostly determined by debrisoquine oxidation phenotype. Following a standard oral dose of the drug, poor metabolizers achieve six times higher plasma concentrations than extensive metabolizers. Poor metabolizers of debrisoquine and metoprolol exhibit abnormal pharmacokinetics of metoprolol, which can result in an overdose after administration of a standard dose [4]. Larrey, et al. described the first and only case of acute non-icteric hepatitis induced by metoprolol. With rechallenge and exclusion of other factors providing convincing evidence of a causal relationship, the authors reasoned that because of an association between debrisoquine oxidation phenotype and susceptibility to liver damage induced by the antianginal agent, perhexiline, the oxidation of metoprolol might be impaired in their patient, leading to toxicity [5]. Lennard, in his commentary on the first reported case, mentions that metoprolol-induced liver injury seemed likely due to an immunological or other idiosyncratic basis and that a relationship to plasma metoprolol concentration was not possibly anticipated [6]. Even though not previously described, we found weak antinuclear antibody positivity with a mitochondrial type of pattern in our patient that became negative after the plasma exchange sessions. Antibodies to the mitochondrial type are of seven types (M1 to M7) and are associated with many conditions: for example, anti-M2, M4, M8, and M9 - primary biliary cirrhosis; M2 - autoimmune hepatitis; M3 - drug-induced lupus erythematosus, and M6 - drug-induced hepatitis [7]. Even though we did not subtype the antibody, antinuclear antibodies, as seen in our patient, can be associated with severe drug-induced liver injury, substantiating Lennard's theory of immune-mediated drug toxicity with metoprolol. Metoprolol use is associated with a low rate of mild-to-moderate elevations of serum aminotransferase levels, usually asymptomatic and transient with resolution with the continuation of therapy - the phenomenon of hepatic adaptation. Liver injury associated with beta-blockers has a latency to onset between two to 12 weeks with a hepatocellular pattern of liver injury, which was evident in our patient. There was a concern regarding the possibility of statin-induced liver injury in our patient as it has been more frequently documented than with metoprolol. However, the number needed to harm (NNH) is one million with statin use, which is still a rare event. The current score-based-diagnostic systems with regards to DILI, even though not precise, are the only methodology by which clinicians can assess causality. RUCAM is the most commonly and widely utilized system in this regard. Based on RUCAM analysis scoring, which is validated and available online, statins scored 5 (possible adverse drug reaction) and metoprolol scored 7 (probable adverse drug reaction in the context of this patient’s disease course); this is why we attributed DILI to metoprolol rather than statins. It has been shown that prolonged latency as seen with most of the types of statins presents with a mixed pattern of liver injury, in contrast to the hepatocellular pattern with autoantibody positivity seen in our patient. The majority of cases resolve once the statins are discontinued, which is contrary to that seen in our patient; the statin was stopped in the early part of November 2016, and clinical and biochemical improvement should have been evident by mid-March 2017. 

Hypersensitivity reactions and autoantibody formation are not reported previously with metoprolol use. In the only reported case of metoprolol-related hepatotoxicity, the liver biopsy showed moderate steatosis and occasional ballooned hepatocyte without inflammation or fibrosis only, possibly reflecting the mild nature of insult early on that improved with drug withdrawal only [5]. In our patient, severe liver injury with jaundice translated to extensive bridging necrosis and mixed neutrophilic and lymphocytic pattern of inflammation with early fibrosis laying down on liver biopsy - features not described with this drug before. We chose plasmapheresis as a treatment modality considering the fact that the auto-antibody was positive and the patient was progressively developing overt hepatic encephalopathy. Plasma exchange has been shown to be useful in the treatment of autoimmune diseases and management of hepatic encephalopathy; it acts by decreasing levels of proinflammatory cytokine storm, toxic endogenous anticoagulants, and end products of oxidative stress, as well as improving anti-inflammatory responses [8-10]. There is very little data supporting the role of therapeutic plasmapheresis in the treatment of DILI. However, only a few causes of DILI have definitive treatment, such as N-acetyl cysteine for paracetamol poisoning, repeated activated charcoal gastric lavage for mushroom poisoning, and liver transplantation for DILI leading to acute liver failure. Plasma exchange is not a definitive or validated treatment for DILI as is the case with any other modality of treatment in this regard, such as steroids, ursodeoxycholic acid, or liver assist devices. However, plasma exchange has been shown to improve clinical outcomes, especially in patients with hepatic encephalopathy. In our patient, there was no definite role of liver transplantation and steroid use as the liver biopsy did not reveal features of autoimmune hepatitis in the latter and halting the drug did not yield instantaneous benefit. Hence, plasma exchange was considered the next best approach to treatment. We did not attempt a rechallenge with the drug, given the severe drug-induced liver injury with the first exposure, and such high-risk re-challenges are currently not recommended. Drug rechallenge, even though a component of RUCAM, is not a requisite for diagnosis of DILI and drug causality in a real life scenario, especially in patients who have had severe drug reactions. Our patient had developed jaundice and encephalopathy, which was a severe grade of liver injury, and rechallenge with the drug had its limitations, including patient and family consent for the same. This was one important reason why we did not perform a re-challenge so as to avoid an anticipated severe life-threatening reaction.

## Conclusions

We present the second case of metoprolol-induced liver injury and the first metoprolol-induced icteric liver injury leading to hepatic encephalopathy in an elderly female managed with plasmapheresis. Our patient also presented with auto-antibody formation possibly associated with severe drug-induced liver injury substantiating the role of immune mechanisms in causing metoprolol-related liver injury. Even though extremely rare, metoprolol should be considered a cause of severe liver injury based on critical patient history analysis and drug causality assessment. Therapeutic plasma exchange could prove highly beneficial early in the disease. 
